# Cr_13_Ni_5_Si_2_-Based Composite Coating on Copper Deposited Using Pulse Laser Induction Cladding

**DOI:** 10.3390/ma10020160

**Published:** 2017-02-10

**Authors:** Ke Wang, Hailin Wang, Guangzhi Zhu, Xiao Zhu

**Affiliations:** National Engineering Research Center of Laser Processing, School of Optical and Electronic Information, Huazhong University of Science and Technology, Wuhan 430074, China; wk900129@sina.com (K.W.); wanghl@hust.edu.cn (H.W.); zgzlaser@hust.edu.cn (G.Z.)

**Keywords:** pulse laser induction cladding, copper, metal silicide, experimental and numerical study, high-temperature wear resistance

## Abstract

A Cr_13_Ni_5_Si_2_-based composite coating was successfully deposited on copper by pulse laser induction hybrid cladding (PLIC), and its high-temperature wear behavior was investigated. Temperature evolutions associated with crack behaviors in PLIC were analyzed and compared with pulse laser cladding (PLC) using the finite element method. The microstructure and present phases were analyzed using scanning electron microscopy and X-ray diffraction. Compared with continuous laser induction cladding, the higher peak power offered by PLIC ensures metallurgical bonding between highly reflective copper substrate and coating. Compared with a wear test at room temperature, at 500 °C the wear volume of the Cr_13_Ni_5_Si_2_-based composite coating increased by 21%, and increased by 225% for a NiCr/Cr_3_C_2_ coating deposited by plasma spray. This novel technology has good prospects for application with respect to the extended service life of copper mold plates for slab continuous casting.

## 1. Introduction

Copper and its alloys are widely used as heat exchangers and conductors. They are also used for other cooling components, such as for copper mold plates for slab continuous casting and in tuyeres used in blast furnaces. This is due to their excellent thermal conductivity. However, the low hardness and the poor wear resistance reduce the service life of the copper components, especially in environments with elevated temperature. In order to extend the service life of the copper components, wear-resistant coatings were prepared on copper by thermal spraying and electroplating techniques. However, these techniques create a non-ideal metallurgical interface between the coating and the substrate, with excessive porosity in the coating [1]. Coatings deposited by laser cladding technology often form metallurgical bonds with substrates. However, owing to the extremely low laser absorptivity to copper, laser surface treatments on copper have rarely been reported, with no practical applications in industry. A two-step laser cladding method was selected by early researchers as the only way to fabricate coatings with good adhesion to copper substrate. Kwok carried out laser surface alloying of copper with Ti by pasting the Ti powders onto copper first [2]. Wang prepared a Ni-Co duplex coating on copper using a pre-electrodeposite Ni/n–Al_2_O_3_ interlayer on a copper substrate first [3]. Two-step laser cladding essentially utilizes the high laser absorptivity of pre-coated layer to absorb laser energy for melting both in substrate and powder beds by heat transference. However, the restricted thickness of coatings prepared by two-step laser cladding and the complicated process limit its practical applications [4,5].

Laser induction hybrid cladding (LIHC) technology was first put forward by Grilloud [6]. Owing to the interaction between laser energy and induction heating, LIHC not only can effectively improve the powder deposition rate but completely avoid cracks in the hardfaced coatings. In the past few years, research on LIHC has been widely carried out. Farahmand deposited a composite coating which was composed of 40% weight fraction of Ni and 60% weight fraction of tungsten carbide (WC) on A36 mild steel by induction-assisted diode laser hybrid cladding [7]. Zhou deposited Ni60A/WC and Fe/WC composite coatings on A3 steel by continuous wave CO_2_ laser induction hybrid cladding [8,9]. To our knowledge, previous research has mostly concentrated on continuous laser (e.g., CO_2_ laser, fiber laser and diode laser) induction hybrid cladding. However, for highly reflective copper, most of the continuous lasers cannot achieve the required energy density for one-step laser surface treatment. Compared with the continuous laser, the pulse laser owns a higher peak power which can output larger laser energy in a short time so that a high energy density can be achieved. The combination of pulse laser power and induction power provides sufficient energy density which ensures the metallurgical bonding between coating and copper substrates.

Cr_13_Ni_5_Si_2_ metal silicide was reported to have outstanding hardness (1080 HV) and wear resistance [10]. More importantly, it showcases an anomalous temperature dependence which means metal silicide can still provide excellent wear resistance performance under elevated temperatures in working environments. Therefore, Cr_13_Ni_5_Si_2_-based metal silicide has attracted the interest of many researchers. Fang prepared short cylinder-like Cr_13_Ni_5_Si_2_ alloy ingot by a copper mold laser melting furnace [11]. Wang deposited Cr_13_Ni_5_Si_2_ metal silicide-based composite coatings on tool steel 9Cr2 substrate by LIHC and analyzed the influences of parameters on microstructures and mechanical properties [12]. The excellent high temperature property makes the Cr_13_Ni_5_Si_2_-based metal silicide an ideal hard coating material on copper for applications in elevated temperatures.

It is a great challenge to deposit a Cr_13_Ni_5_Si_2_-based hard coating on copper using pulse laser induction hybrid cladding (PLIC) without any cracks. The well-known high thermal conductivity of copper and the inherent periodic output characteristic of pulse laser aggravate the difficulty of depositing crack-free coating. In addition, the mechanisms as to how the continuous laser induction cladding reduces the cracking of the coatings were interpreted based on numerical analysis [13]. Hence, theoretical analysis about the temperature evolution characteristics and corresponding crack behavior in PLIC should be established.

In this paper, a Cr_13_Ni_5_Si_2_-based composite coating was successfully deposited on copper by PLIC, aiming at improving the high-temperature wear resistance of copper components. Microstructure and phases present were analyzed using scanning electron microscopy and X-ray diffraction. The wear properties of the Cr_13_Ni_5_Si_2_-based composite coating were evaluated under room temperature and at 500 °C. A finite element model was established for analyzing and comparing the difference of temperature evolution associated with crack behaviors in pulse laser cladding (PLC) and PLIC.

## 2. Experimental Details

### 2.1. Experimental Equipment and Materials

**Figure 1:**
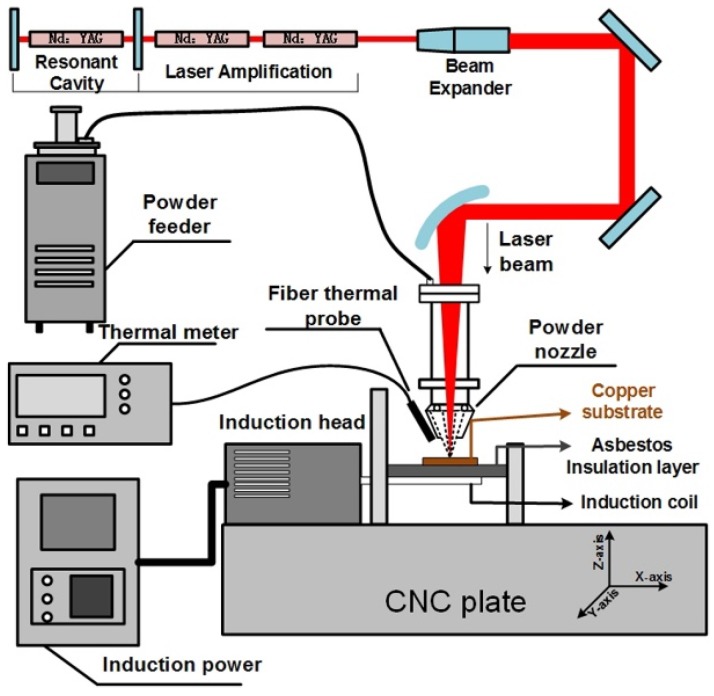
The schematic illustrating of pulse laser induction hybrid cladding (PLIC) system.

Figure 1 shows the schematic illustrating of a pulse laser induction hybrid cladding system. The experimental laser system was our laboratory-made Nd:YAG pulse laser. The laser can reach an average power of 1 kW and a maximum pulse peak power of 10 kW. As a heating source, 60-kW high-frequency induction heating equipment was introduced. The whole PLIC process was carried out in an environment filled with high-purity argon gas. The heating and the insulation processes were supervised using an optical fiber thermometer.

Commercial T2 copper samples with dimensions of 30 mm × 30 mm × 6 mm were selected as the substrates. The raw powders designed for depositing the Cr_13_Ni_5_Si_2_-based composite coatings were prepared using commercially pure chromium, nickel, and silicon elemental powders, with an average particle size ranging from 90 to 150 μm and a nominal chemical composition (wt %) of 50Cr-42.6Ni-6.8Si. The elemental powders were mixed in a ball milling machine for 2 h.

Copper has poor wettability with many other materials, which makes the fabrication of coatings with metallurgical bonding on Cu substrate more difficult [14]. It has been proven that directly depositing Ni–Cr–Si coating on copper is difficult because of the poor wettability between copper substrate and Ni–Cr–Si powders. Monel400 alloy powder has a good wettability with Cu substrate considering it is mainly composed of Cu and Ni, and since the Cu-Ni forms solid solutions in all proportions. Thus, a transition layer measuring 100–200 μm in thickness was deposited on Cu substrate using Monel400 alloy powders with particle size ranging from 90 to 150 μm , which enhance metallurgical bonding between the Cu substrate and Ni–Cr–Si composite coating. Before the PLIC process, the powders were pre-dried for 120 min at 150 °C and the Cu substrate was sand blasted and ethanol rinsed.

### 2.2. Sample Preparation

The Cu substrate was placed on an adiabatic asbestos board which can reduce heat loss. During the PLIC process, firstly, the Cu substrate was heated to a preset temperature by induction heating with a high power. Then the preset temperature was kept with a low power. Meanwhile, the coaxial powder feed laser cladding process started. The process of PLIC is complex as many parameters are involved. In particular, laser pulse duration and repetition rate have great effects on the bonding strength and deposition efficiency of coatings (in order to take the best advantage of pulse energy while ensuring the safety of laser equipment, the product of pulse duration and repetition rate should be 120). Under the condition of stable laser power output, a relatively long pulse duration can obtain higher single pulse energy, which ensures good bonding strength between coating and the substrate. However, a long pulse duration limits the pulse repetition rate, which reduces the deposition efficiency of powders. Therefore, it is necessary to find a balance between laser pulse duration and repetition rate. Through orthogonal experiments, Table 1 shows the optimized parameters of depositing a Monel400 transition layer and a Cr_13_Ni_5_Si_2_ layer. Pulse duration of 6 ms and induction heating at 500 °C were selected as appropriate parameters for depositing one Monel400 transition layer on Cu substrate. Then, four layers Cr_13_Ni_5_Si_2_ composite coatings were deposited on the Monel400 transition layer using a 6-ms pulse duration laser. The induction heating temperature was set at 750 °C when depositing Cr_13_Ni_5_Si_2_ composite coatings for avoiding the production of cracks.

### 2.3. Analysis Methods

Microstructural and compositional analyses were performed using a scanning electron microscope (SEM, ZEISS Sigma HD, Oberkochen, Germany) equipped with an energy dispersive X-ray spectrometer (EDS, OXFORD-X-Max, Oxfordshire, Britain) for analyzing cross-sections of the samples which were polished and etched (HNO_3_-HCl-H_2_O water solution with volume ratio of 1:1.6:3.2). Phase identification was conducted using an X-ray diffractometer (XRD, X’pert Pro, PANalytical, Almelo, Holland) with a Cu target.

The hardness profiles along the depths of the composite coatings were measured by Vickers microhardness tester (430SVD, Wilson hardness, Buehler, Lake Bluff, IL, USA) with an applied load of 100 g and a dwell time of 15 s.

The wear properties of Cr_13_Ni_5_Si_2_ composite coatings at room temperature and 500 °C were evaluated using a ball-on-plate dry sliding tribometer (Bruker UTM-Tribolab, USA). A Si_3_N_4_ ball with a diameter of 6.350 mm (1500 HV–1600 HV) slid on the surface of Cr_13_Ni_5_Si_2_ coatings with a reciprocating motion. Unlike most of the metal material, there is almost no mechanical property degradation of the Si_3_N_4_ ceramic grinding ball under 1200 °C, which makes the test results more reliable and comparable under conditions of elevated temperature. The testing load was 45 N. The relative velocity between the grinding ball and specimen was 10 mm/s with the one-way distance of 10 mm. The reciprocating wear test lasted for 3 h with a total wear sliding distance of 108 m. Copper samples coated with a 0.3-mm-thick NiCr/Cr_3_C_2_ wear-resistant coating using plasma spray method, which had been cut from commercial copper mold plate, were selected as the reference samples. Wear volume losses of the specimens were precisely measured by laser scanning confocal microscopy (LSCM, VK-X200K, KEYENCE, Itasca, IL, USA).

The thermal diffusivities (α) of Cr_13_Ni_5_Si_2_ coatings were measured using a Laser flash diffusivity apparatus (LFA 427, NETZSCH, Selb, Germany) at 50 °C, 210 °C and 400 °C. The specific heat (*C*_p_) was measured as a function of temperature using differential scanning calorimetry (DSC, STA449F3, NETZSCH, Selb, Germany).

## 3. Results and Discussion

### 3.1. Macrostructure of Composite Coating

**Figure 2:**
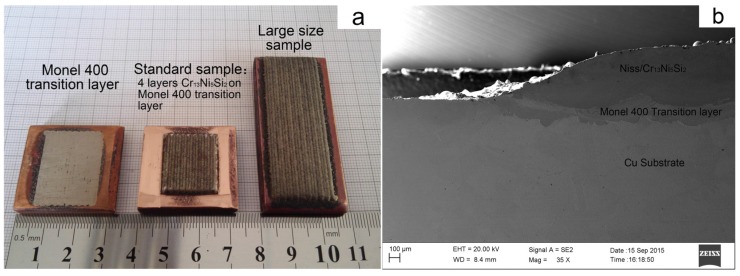
Macro-appearance of samples by PLIC: (**a**) Monel400 transition layer and Cr_13_Ni_5_Si_2_-based composite coating; (**b**) cross-sectional view of the coating.

Depositing Cr_13_Ni_5_Si_2_-based composite coating on copper is difficult because of the low laser absorptivity, high thermal conductivity of copper and poor wettability between two materials. Fortunately, due to the use of PLIC technology and the introduction of the Monel400 transition layer, a defect-free Cr_13_Ni_5_Si_2_-based composite coating was successfully deposited on copper. Figure 2 shows the surface appearance and cross-section morphology of samples deposited by PLIC. In this section, the macrostructure of composite coating was discussed with respect to two aspects: crack behavior and metallurgical bonding.

#### 3.1.1. Crack Behavior in PLC and PLIC

**Figure 3:**
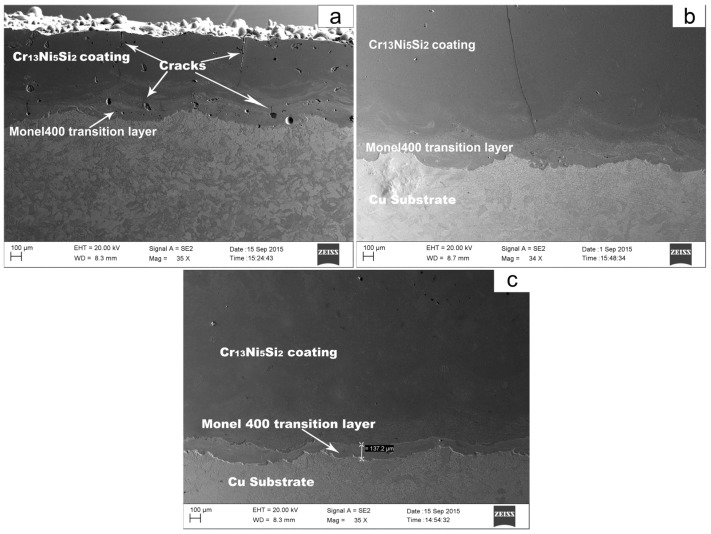
Scanning electron microscope (SEM) images of cross-section crack patterns of coatings deposited using (**a**) pulse laser cladding (PLC); (**b**) pulse laser induction cladding (PLIC) at 600 °C and (**c**) PLIC at 750 °C.

Laser cladding has the characteristics of rapid heating and solidification. Thus, for a certain material system, the production of cracks in coatings can be the result of two causes: the rapid temperature change in space (high thermal gradient) which generates high thermal stress, and the rapid temperature change in time (fast cooling rate) which restrains plastic deformation in rapid solidification. Copper has much higher thermal conductivity than most metal materials, which means that a larger temperature gradient and a faster cooling rate exist during the solidification process in laser cladding on copper substrate. Additionally, compared with continuous laser, pulse laser has a high peak power which can generate a large amount of laser energy in a short time, but a long pulse interval exists between the two laser pulses (for our process, there are 6 ms of laser output but 44 ms without laser output in a pulse period) which produces a larger thermal gradient and a faster cooling rate during the solidification process. As shown in Figure 3a, several cracks were observed in the Cr_13_Ni_5_Si_2_-based composite coating which was deposited on the Monel400 transition layer using PLC.

Brücknera mentioned that additional heat source which follows the laser beam can hold the temperature temporarily at a sufficiently high level in order to enable the equalization of temperature at a level where plastic flow is able to prevent the evolution of large stresses [15]. Wang proposed that the cooling rates decreased greatly due to the effect of the preheating and the post-heat effect of the induction. As a result, the residual stress in the composite coatings decreased greatly [16]. Crack behavior in our experiments indicated that induction heating-assisted pulse laser cladding can reduce or even eliminate cracks. As shown in Figure 3b, one crack was observed when the composite coating was deposited by PLIC with induction heating at 600 °C. However, a composite coating deposited by PLIC with induction heating at 750 °C was free of cracks (see Figure 3c).

#### 3.1.2. Finite Element Modeling

In order to investigate how the induction heating helps to prevent cracking, temperature evolutions in one laser pulse period and multiple laser pulse periods in PLIC and PLC were analyzed and compared using the finite element analysis method.

**Figure 4:**
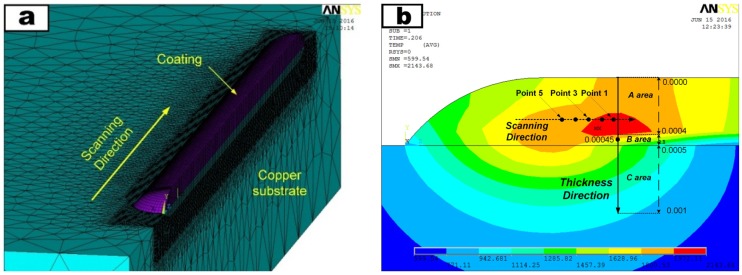
(**a**)The finite element model for single-track coating on Cu substrate; (**b**) thickness direction path and five discrete points along scanning direction.

The Monel400 transition layer had little effect on temperature evolution for it was only 100–200 μm thick and had strong mutual dilution with copper substrate. Therefore, a finite element model for single-track Cr_13_Ni_5_Si_2_ coating (deposited using eight periods of pulse laser outputs) on a copper substrate was built based on ANSYS software (see Figure 4a). In this paper, we adopted a 6-ms pulse width with a 20-Hz repetition rate as pulse laser parameters to analyze the temperature evolution in one laser pulse period along the thickness direction (*y*-direction) and the temperature evolution in multiple laser pulse periods in the scanning direction (*z*-direction) (see Figure 4b). The element “birth and death” was used to model the pulse mode cladding process. In the element “birth and death” technique, the elements of the cladded layer were predefined as inactive elements and during the process of laser pulse output, the elements in this laser interaction area were set to be active. Considering the short interaction time of the pulse laser and rapid heat dissipation through copper substrate, the laser volumetric heat source *Q*_laser_ (w/m^3^) was simulated by moving elliptical heat flux model by using the following equation [17].

(1) $$Q_{\text{laser}}(x,y,z,t)=\{\begin{array}{cc} \frac{6\sqrt{3}\cdot W}{abc\pi \sqrt{\pi }}e^{-3x^{2}/a^{2}}e^{-3y^{2}/b^{2}}e^{-3{\left[z+v\left(\tau -t\right)\right]}^{2}/c^{2}}, & t\in T_{\text{pulse output}}\\ 0, & t\in T_{\text{pulse interval}} \end{array}$$

(2) $$w=\gamma \cdot P_{\mathrm{peak}}=\gamma \cdot \frac{P_{\mathrm{Average}}}{f\cdot \Delta t}$$

where *a*, *b*, *c*, are the respective radii of the ellipsoid, *v* is the laser scanning speed, *P*_average_ (850 w) is laser average power, and *f* (20 Hz) and Δ*t* (6 ms) are the laser repetition rate and pulse duration, respectively. While the Monel400 transition layer has little effect on temperature evolution, it improves the laser absorptivity (γ = 0.38). The piecewise function in Equation (1) reflects the heat source loading characteristic in a laser pulse period. Circulation commands based on ANSYS APLD language were used to perform the periodic loading of multiple laser pulses. The activation process in the element “birth and death” technique which reflects the forming process of coating was completely synchronized with periodic loading of the pulse laser heat source, which is close to the real pulse-mode cladding process.

**Figure 5:**
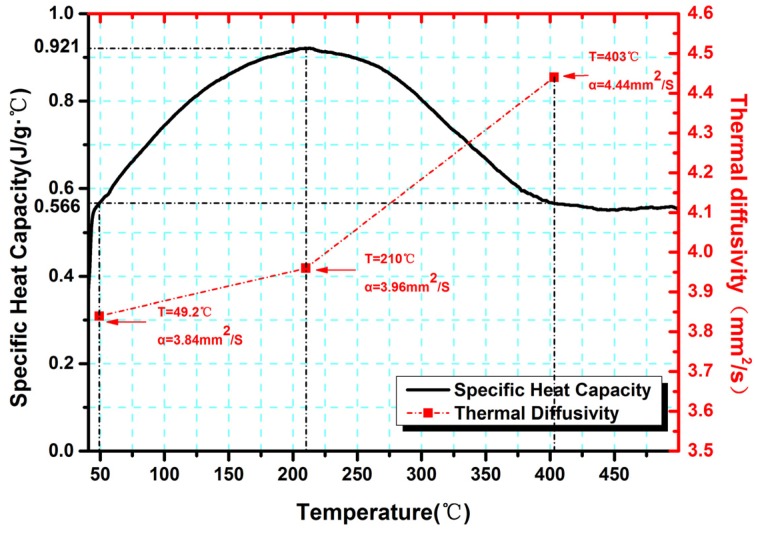
The specific heat of Cr_13_Ni_5_Si_2_-based composite coatings and the thermal diffusivity at 50 °C, 210 °C and 400 °C.

The initial temperature of the substrate is expressed as:

(3) $$T\left(x,y,z,0\right)=T_{i}$$

where *T*_i_ is the initial temperature of the substrate before starting the cladding process. In terms of PLIC, *T*_i_ is equal to the induction temperature (i.e., 600 °C or 750 °C). As for PLC, *T*_i_ is equal to the ambient temperature (25 °C). The temperature-dependent material properties, such as thermal diffusivities and specific heat for the Cr_13_Ni_5_Si_2_-based coating, are shown in Figure 5. The melting point of the Cr_13_Ni_5_Si_2_-based coating in our experiment is 1392 °C by TG/DTA test.

#### 3.1.3. Temperature Evolution in PLC and PLIC

**Figure 6:**
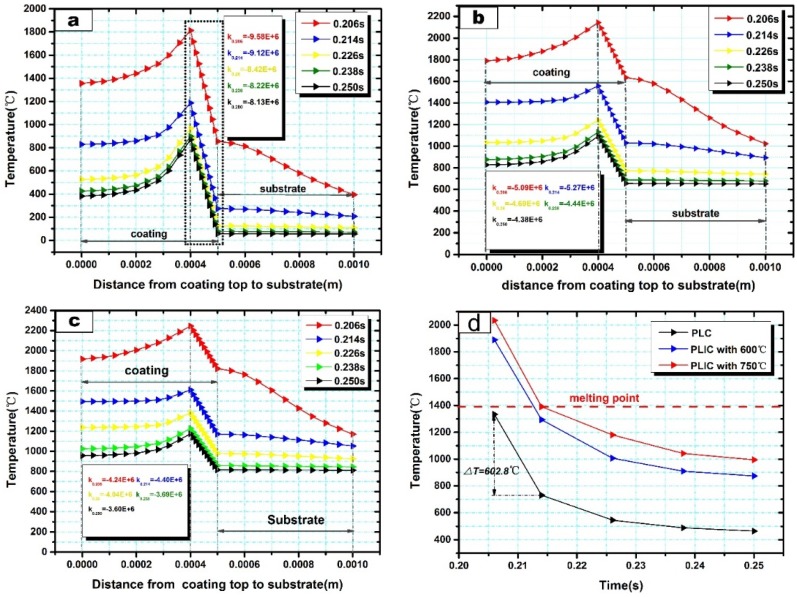
Thickness-direction temperature evolution during the fifth laser pulse interval in (**a**) PLC and PLIC at (**b**) 600 °C and (**c**) 750 °C, (**d**) the temperature–time curve at 0.206–0.250 s at a point with position of *y* = 0.00045 in the thickness direction.

Unlike the laser output characteristic of continuous laser, for pulse laser, a pulse interval exists between two laser pulses. Therefore, temperature evolution characteristic in a pulse interval is an essential part of pulse laser processing. The fifth pulse interval (0.206 s–0.250 s, when the fifth laser pulse had completed the output at 0.206 s and the sixth laser pulse was to output at 0.250 s) was selected to study the temperature evolutions. Due to the short interaction time of the one-period laser pulse, temperature evolutions along the thickness direction (*y*-direction, from *y* = 0 to *y* = 0.001, Figure 4b) were mainly considered. Figure 6a–c presents the simulation results of temperature evolutions in PLC and PLIC at 600 °C and 750 °C. Each curve indicates the temperature distribution from the top of the coating (*y* = 0) to the Cu substrate (*y* = 0.001 m) at a certain time in the fifth pulse interval (0.206 s, 0.214 s, 0.226 s, etc.). The result reveals that the maximum temperatures are all located around 0.0004 m below the coating surface because of the heat convection with air and heat conduction with copper substrates. A drastic linear temperature reduction occurs in the lower regions of coating (from *y* = 0.0004 m to *y* = 0.0005 m) because of the high thermal conductivity of the copper substrates. The rapid temperature reduction in this region increases the possibility of cracking. Farahmand also mentioned that the area close to the clad/substrate interface has the highest tensile stress in the thickness direction [13]. We calculated the slope (*k*) in this regions with Equation (4),

(4) $$k=\frac{T_{0.0005}-T_{0.0004}}{0.0005-0.0004}$$

As temperature reduction in this regions shows a linear decline, it is possible to describe the temperature gradient with the absolute value of the slope. Compared to PLC in Figure 6a, the absolute values of the slope are much smaller in PLIC at 600 °C (see Figure 6b) and 750 °C (see Figure 6c). Temperature gradients of PLC are twice as large as PLIC at 750 °C in these regions. The introduction of induction heating greatly reduces the temperature gradient in the lower region of the composite coating, which reduces the thermal stress.

Furthermore, as the thermal stress is mainly concentrated in the lower region of the composite coating, it is necessary to analyze the cooling rate during the laser pulse interval, which is important to the stress release by plastic deformation. Figure 6d shows the cooling curve in the fifth pulse interval at a point with position of *y* = 0.00045 (see Figure 4b). Interestingly, during the fifth laser pulse interval, the temperature evolution trends are similar in PLC and PLIC at 600 °C and 750 °C. Moreover, it is worth noting that a relatively large cooling rate exists at the beginning of the laser pulse interval (between 0.206 s and 0.214 s) in all cases. Remarkably, in PLC, this rapid cooling happens below the melting point which means there is not enough time for producing plastic deformation and stress release. However, in PLIC at 750 °C, the induction heating raises the temperature above the melting point between 0.206 s and 0.214 s, which prevents the composite coatings from suffering the rapid cooling process in solid state. The crack can not be completely eliminated when the induction heating is inadequate to assure the whole rapid cooling process of coating in liquid state (e.g., in PLIC with 600 °C).

**Figure 7:**
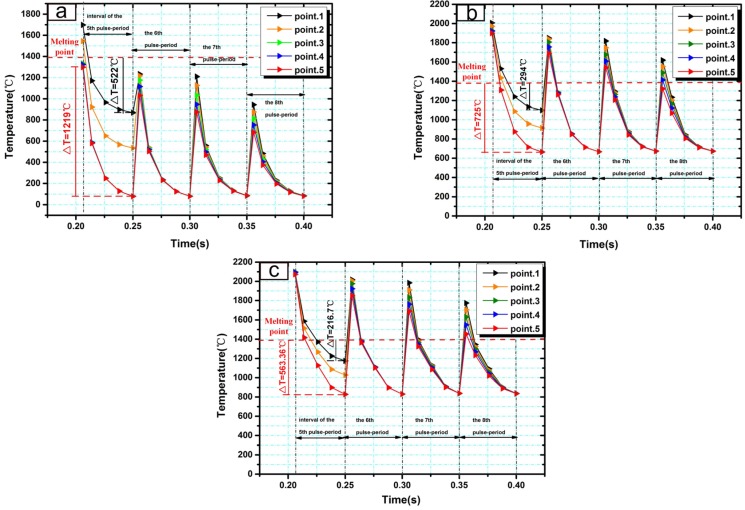
Temperature evolution of five discrete points along scanning direction in (**a**) PLC and PLIC at (**b**) 600 °C and (**c**) 750 °C during the fifth laser pulse interval and the subsequent three laser pulse periods.

For multiple laser pulse periods, temperature evolutions along scanning direction (*z*-direction) were mainly considered. Five discrete points around the interaction region of the fifth laser pulse were selected to study the temperature evolution along the scanning direction (see in Figure 4b). Temperature evolutions during the fifth laser pulse period and the next three pulse periods were simulated. As the thermal stress exists only in solids, the temperature changes associated with thermal stress should be discussed under the melting point. Figure 7a shows the temperature evolutions of the five discrete points in PLC. Large temperature differences exist in adjacent points because of the laser rapid solidification. During the fifth pulse interval, temperature-drop values associated with thermal stress were 522 °C at point 1 (the difference between the melting point and the temperature at 0.250 s) and 1219 °C at point 5 (the difference between the highest temperature at 0.206 s and the temperature at 0.250 s). Based on Hooke’s law, the temperature-drop value determines the deformation when there is no space constraint. Different temperature-drop values at two points cause the difference in the deformations. In laser rapid solidification, different temperature-drop values at two points produce stress because the space constraint truly exists. The difference between two temperature-drop values at point 1 and point 5 was 697 °C (1219 °C − 522 °C = 697 °C) which can reflect the the magnitude of thermal stress to a certain extent. As shown in Figure 7b–c, in PLIC at 600 °C and 750 °C, the differences of temperature-drop values at point 1 and point 5 are 431.5 °C and 346.6 °C during the fifth pulse interval, which are much lower than the values in PLC. The introduction of induction heating greatly decreases the difference of temperature-drop values between adjacent points in laser pulse interval, which reduces the thermal stress in the coating layer and avoids the cracking of coating. As Figure 7a–c shows, the temperature changes of these five points become synchronized during the subsequent laser pulse periods in both PLC and PLIC at 600 °C and 750 °C. However, the introduction of induction heating decreases the cooling rate of coating during the subsequent laser pulse periods, such as in the sixth laser pulse period, when the cooling rates of PLC and PLIC at 600 °C and 750 °C were 4453 °C/s, 2696 °C/s and 2180 °C/s, respectivly.

#### 3.1.4. Metallurgical Bonding

**Figure 8:**
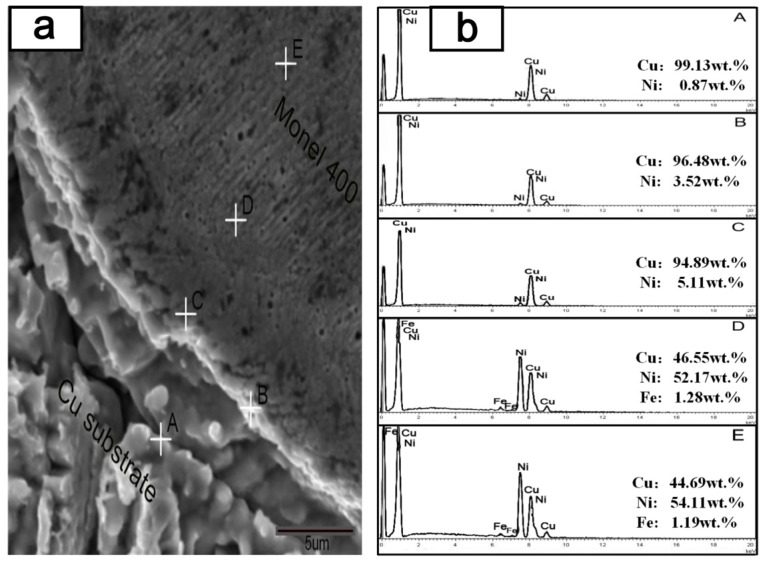
Interface zone of Cu substrate and Monel400 transition layer and EDS results of different areas.

Good metallurgical bonding should form between coatings and substrates, otherwise flaking of the coating could easily occur under working conditions. In particular, for components like copper mold plates which are working under elevated temperature, the metallurgical bonding between coatings and Cu substrate should be primarily investigated. Figure 8 shows the SEM image of the interface zone between Cu substrate and Monel400 transition layer as well as the EDS results of chemical composition in different regions of the interface zone. Point A is on the Cu substrate with the highest content of Cu, and the content of Cu decreases gradually from point A to point E. Meanwhile, the content of Ni, which only exists in the Monel400 alloy, increases gradually. In the region of point E, the content of Cu is 44.69 wt % which is higher than the content of Cu in Monel400 powders (around 35 wt %), which indicates the dilution still exists in point E. The Cu substrate and the Monel400 transition layer, which were mutually soluble at the interface, constituted the metallurgical bonding.

**Figure 9:**
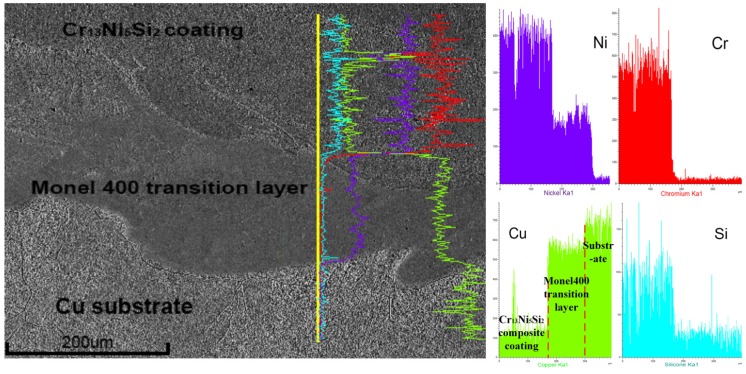
Line scan result of the Cu substrate–Monel400 transition layer–Cr_13_Ni_5_Si_2_ composite coating.

Figure 9 shows the line scan result of the Cu substrate–Monel400 transition layer–Cr_13_Ni_5_Si_2_ composite coating. The copper element should not be found in the Cr_13_Ni_5_Si_2_-based composite coating. However, a certain amount of copper was found in the Cr_13_Ni_5_Si_2_-based composite coating close to the interface of Monel400 transition layer–Cr_13_Ni_5_Si_2_ composite coating, which proves the metallurgical bonding between them.

### 3.2. Microstructure of Composite Coating

**Figure 10:**
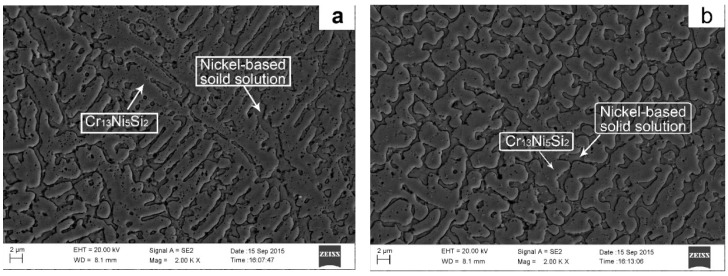
Microstructures of the Cr_13_Ni_5_Si_2_-based composite coating (**a**) in lower regions of coating and (**b**) in upper regions of coating.

**Figure 11:**
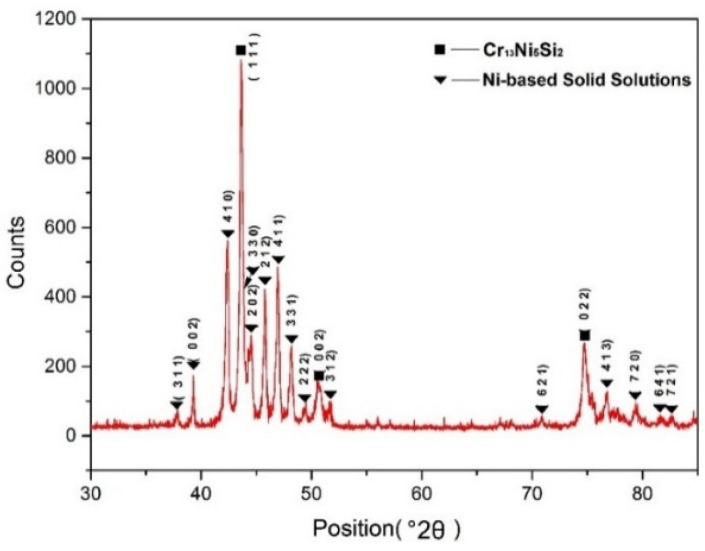
XRD spectra of the Cr_13_Ni_5_Si_2_ composite coating.

Figure 10 shows the SEM images of cross-sectional microstructure of composite coating. There is a primary phase with a dendritic morphology and another phase that forms in the interdendritic regions. As shown in Figure 11, the XRD result indicates that the main constituent phases in the composite coating were Cr_13_Ni_5_Si_2_ ternary metal silicide and nickel-base solid solution. The EDS result indicates that the average chemical composition (wt %) of the dendritic primary phase is approximately 5.7Si-36.6Ni-57.7Cr and is identified as Cr_13_Ni_5_Si_2_ primary phase. The phase in the interdendritic regions has a composition of 13.7Si-46.5Ni-39.8Cr and is confirmed to be the nickel-base solid solution. The Cr_13_Ni_5_Si_2_ primary phase has intrinsic high hardness, however, the pure Cr_13_Ni_5_Si_2_ is so brittle that it has little significance for practical applications. Depositing a ductile metal solid solution-toughened Cr_13_Ni_5_Si_2_ alloy is an effective way to enhance its toughness and ductility [18]. The nickel-base solid solutions in interdendritic regions act as the toughening phase.

Figure 10a shows typical columnar dendrites in first layer of the Cr_13_Ni_5_Si_2_-based composite coating which was close to substrate. It is well-known that solidification in laser melting processes is epitaxial and columnar dendrites grow in different directions paralleling to the heat transfer direction [19]. Despite the fact that induction heating reduces the thermal gradient, the high thermal conductivity of copper strengthens the directional solidification during the process of depositing the first layer of the Cr_13_Ni_5_Si_2_-based composite coating. When depositing the subsequent layers, the thermal gradient in the melt pool becomes lower because of the heat accumulation by overlapped tracks and layers and the continuous heat production by induction heating. Far away from the high thermal conductivity of the copper substrate as well as the low thermal gradient in the melt pool, the directional solidification becomes weak. Consequently, finer dendrites with disorientated microstructures are formed in upper regions of the Cr_13_Ni_5_Si_2_ composite coating in Figure 10b.

### 3.3. Hardness and Wear Resistance

**Figure 12:**
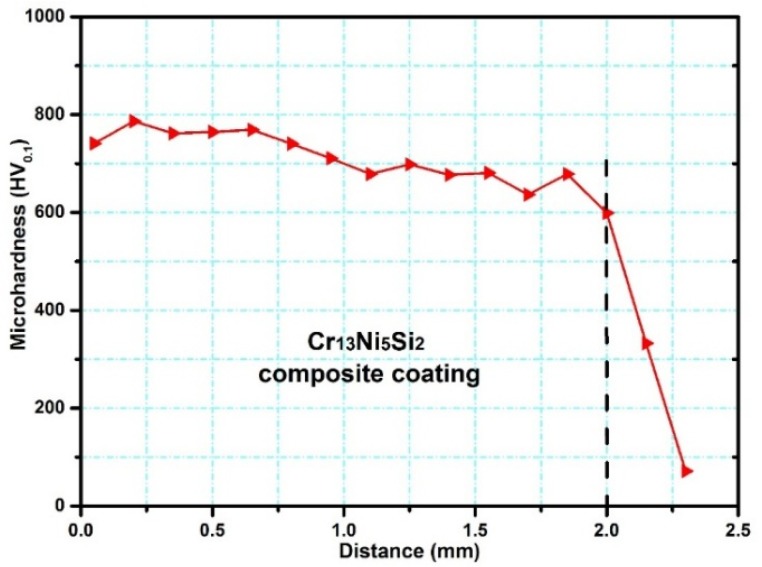
Vickers microhardness curve along the depth direction.

Figure 12 shows that the distribution of microhardness along the depths of the direction was uniform (760 ± 20 HV) in the upper regions of the Cr_13_Ni_5_Si_2_-based composite coating.

**Figure 13:**
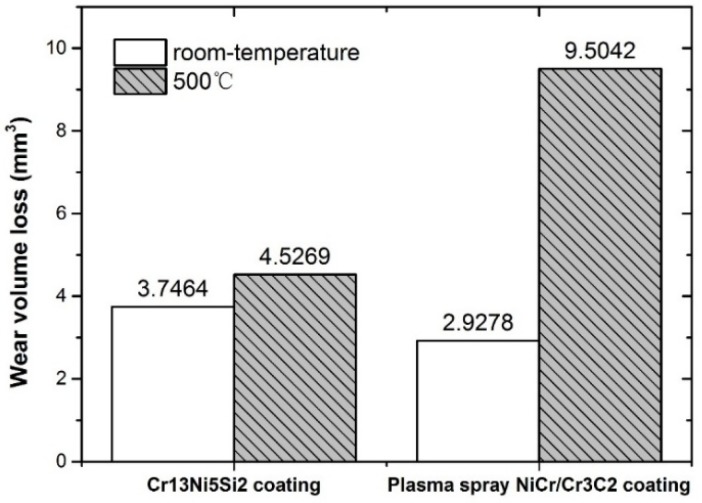
Wear volume loss of the Cr_13_Ni_5_Si_2_-based composite coating and the plasma spray NiCr/Cr_3_C_2_ coating at room temperature and at 500 °C.

Figure 13 shows the wear volume loss of the Cr_13_Ni_5_Si_2_-based composite coating and reference samples in room temperature and dry sliding wear tests at 500 °C. In room temperature wear testing, due to the relevant higher hardness of the NiCr/Cr_3_C_2_ coating (1035 HV), the wear volume of reference sample was even lower than that of the Cr_13_Ni_5_Si_2_-based composite coating. However, in the wear test at 500 °C, the wear volume of the NiCr/Cr_3_C_2_ coating greatly increased by 225% as compared with wear volume at room temperature. The NiCr/Cr_3_C_2_ coating deposited by plasma spray is only 0.3-mm thick and has a poor mechanical bonding with the substrate, therefore, the coating is more likely to fall off in the wear test at 500 °C. Additionally, the high porosity of the NiCr/Cr_3_C_2_ coating deposited by plasma spray is also an important factor that decreases the wear resistance in the wear test at 500 °C. Because of the good metallurgical bond with the copper substrate, the compact microstructure and the anomalous hardness–temperature relationship of the Cr_13_Ni_5_Si_2_ composite coating, the wear volume increased by 21% as compared with the wear test at room temperature.

**Figure 14:**
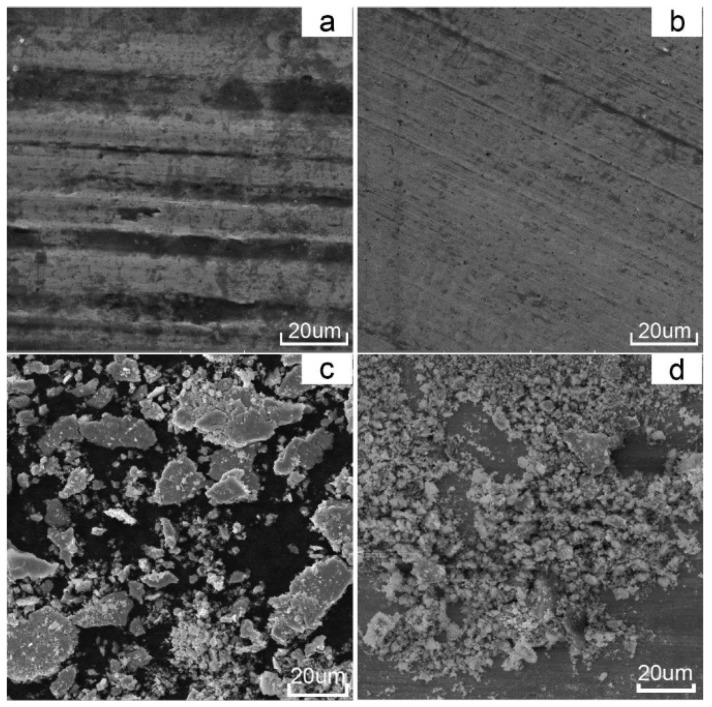
Worn surface micrographs of the Cr_13_Ni_5_Si_2_-based composite coating at (**a**) room temperature and (**b**) 500 °C; and wear debris at (**c**) room temperature and (**d**) 500 °C.

**Figure 15:**
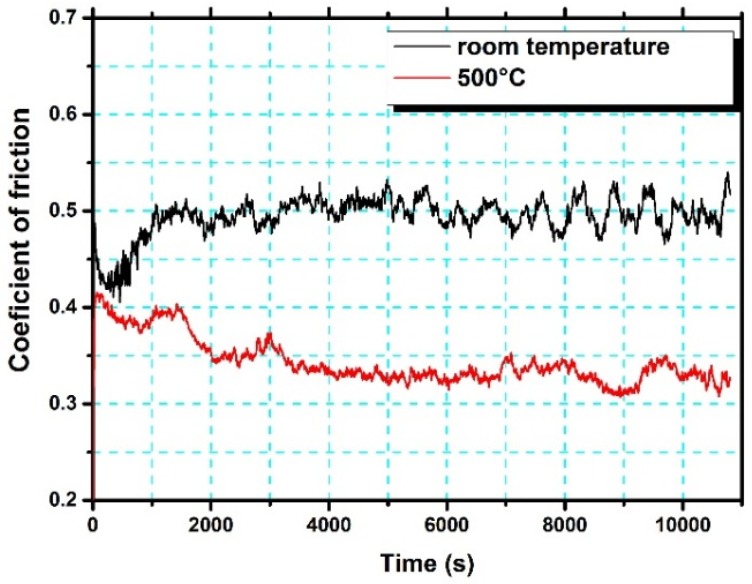
Coefficient of friction as a function of time for the Cr_13_Ni_5_Si_2_ coating and the Si_3_N_4_ counterpart ball at room temperature and at 500 °C.

The worn surfaces and debris of the Cr_13_Ni_5_Si_2_-based composite coatings were analysed with a view to understanding the wear mechanisms at room and elevated temperatures. As shown in Figure 14, the worn surfaces of the Cr_13_Ni_5_Si_2_-based composite coatings after wear tests at both room temperature and 500 °C show no adhesive wear characteristics. However, some micro-plough grooves can be observed in both wear tests, which is considerated as clear evidence of abrasive wear. It is widely accepted that the abrasive wear resistance is mainly dependent on the material hardness. Generally, for most metal materials, abrasive wear should be more serious at elevated temperatures as the hardness typically decreases with the increasing temperature. However, it is worth noting that the micro-plough grooves on the worn surface of the wear test at 500 °C in Figure 14b were tinier and shallower than on the worn surface of the room temperature wear test in Figure 14a. The morphologies of the wear debris in room temperature wear test were primarily large and flake-like (Figure 14c), while a great quantity of fine granular debris and some flake-like debris was observed in the wear test at 500 °C in Figure 14d. As shown in Table 2, EDS analysis indicated that surface oxidation in the wear tests 500 °C was more intense. At room temperature, the slight surface oxidation was mainly caused by friction heat production, and the chemical composition of both the worn surface and the large flake-like debris was much closer to the original composition of the coating. Therefore, excluding adhesive wear and slight surface oxidation, the rough and deep micro-plough grooves on the coating surface in the wear test at room temperature were mainly caused by two-body abrasion between the Cr_13_Ni_5_Si_2_-based composite coatings and the Si_3_N_4_ counterpart ball. EDS results show that the worn surface and the fine granular debris in the wear test at 500 °C were highly enriched in O and Ni with a little amount of Cr and Si, which means that nickel oxide-dominated complex oxide film was formed on the coating surface in the wear test at 500 °C. Inman mentioned that the “glaze” oxide surface formed by nickel oxide was highly wear protective [20]. Figure 15 shows that the formation of this complex oxide film obviously reduced the coefficient of friction, which proves the positive effect of this oxide film as wear protective. The oxide film prevents the Si_3_N_4_ counterpart ball from directly microploughing on the coating surface. Instead, the fine and straight parallel grooves in the wear test at 500 °C indicated that the fine granular oxide debris was acting as an abrasive agent between the oxide film and the Si_3_N_4_ counterpart ball. Therefore, wear loss in the wear test at 500 °C was mainly caused by falling oxide debris in a reciprocating motion between the Si_3_N_4_ counterpart ball and the oxide film and the abrasion by microploughing between the oxide debris and the oxide film.

## 4. Conclusions

In this study, a Cr_13_Ni_5_Si_2_-based metal silicide composite coating was deposited on pure copper using PLIC technology. Good metallurgical bonding between the Cr_13_Ni_5_Si_2_-based composite coating and the copper substrate was obtained due to the introduction of the Monel400 transition layer.

Induction heating greatly slows down the temperature gradient in the lower region of composite coating, and heats the temperature above the melting point, which prevents the composite coatings from experiencing the rapid cooling process in a solid state at the beginning of pulse interval. The induction heating also greatly decreases the difference of temperature-drop values between adjacent areas, which reduces the thermal stress in coating.

Compared with the room temperature wear test, the wear volume of Cr_13_Ni_5_Si_2_-based composite coating in the wear test at 500 °C increased by 21% (and increased by 225% for the NiCr/Cr_3_C_2_ coating deposited by plasma spray). Nickel oxide-dominated complex oxide film was formed on the coating surface in the wear test at 500 °C, which prevented the Si_3_N_4_ counterpart ball from directly micro-ploughing on the coating surface.

## Figures and Tables

**Table 1:** Optimized parameters for PLIC in the investigation.

Parameters	Monel400 Transition Layer	Cr_13_Ni_5_Si_2_-Based Composite Coating
Induction temperature	500 °C	750 °C
Pulse duration (Δ*t*)	6 ms	6 ms
Pulse repetition rate (*f*)	20 Hz	20 Hz
Laser average power (*P*_average_)	780 W	850 W
Beam diameter	1.2 mm	1.2 mm
Scan speed	510 mm/min	400 mm/min
Powder feed rate	11.8 g/min	16.55 g/min

**Table 2:** EDS results of the worn surface and the wear debris of the Cr_13_Ni_5_Si_2_ coating.

Analysis Object	Chemical Composition (wt %)			
	Cr	Ni	Si	O
Worn surface at room-temperature	50.71	34.24	4.55	10.5
Worn surface at 500 °C	9.25	40.54	5.45	44.76
Debris in room-temperature wear test	46.13	36.89	2.87	14.12
Debris in the wear test at 500 °C	9.44	41.48	4.6	44.48
